# C-S-H Pore Size Characterization Via a Combined Nuclear Magnetic Resonance (NMR)–Scanning Electron Microscopy (SEM) Surface Relaxivity Calibration

**DOI:** 10.3390/ma13071779

**Published:** 2020-04-10

**Authors:** Christoph Naber, Florian Kleiner, Franz Becker, Long Nguyen-Tuan, Christiane Rößler, Merlin A. Etzold, Jürgen Neubauer

**Affiliations:** 1 GeoZentrum Nordbayern, Mineralogy, University of Erlangen-Nuernberg (FAU), Schlossgarten 5a, 91054 Erlangen, Germany; franz.becker@fau.de (F.B.); juergen.neubauer@fau.de (J.N.); 2 F. A. Finger-Institute for Building Materials Science, Bauhaus-University Weimar, Coudraystraße 11A, 99423 Weimar, Germany; florian.kleiner@uni-weimar.de (F.K.); long.nguyen.tuan@uni-weimar.de (L.N.-T.); christiane.roessler@uni-weimar.de (C.R.); 3 Department of Applied Mathematics and Theoretical Physics, University of Cambridge, Wilberforce Road, Cambridge CB3 0WA, UK; mae38@cam.ac.uk

**Keywords:** nuclear magnetic resonance (NMR), scanning electron microscopy (SEM), pore size distribution, calcium silicate hydrate, tricalcium silicate

## Abstract

A new method for the nuclear magnetic resonance (NMR) surface relaxivity calibration in hydrated cement samples is proposed. This method relies on a combined analysis of 28-d hydrated tricalcium silicate samples by scanning electron microscopy (SEM) image analysis and ^1^H-time-domain (TD)-NMR relaxometry. Pore surface and volume data for interhydrate pores are obtained from high resolution SEM images on surfaces obtained by argon broad ion beam sectioning. These data are combined with *T*_2_ relaxation times from ^1^H-TD-NMR to calculate the systems surface relaxivity according to the fast exchange model of relaxation. This new method is compared to an alternative method that employs sequential drying to calibrate the systems surface relaxivity.

## 1. Introduction

The nature of the pore space in hydrated cement is essential for the buildup of final strength in concrete constructions. Fewer and smaller pores in concrete lead to higher compressive strength. The pore space is difficult to access in its entirety due to the wide range of length scales starting from nanometer size interlayer and gel pores in C-S-H, to interhydrate pores between individual hydrate phases, and micrometer size capillary pores. Commonly used techniques to measure the pore volume, like mercury intrusion porosimetry or gas adsorption, are not sensitive to C-S-H porosity [1]. Furthermore, they require a water removal step prior to the measurement. This can induce artifacts, especially for interlayer and gel pores depending on the drying method used [2].

In contrast, no pore water is removed during sample preparation prior to measurements employing ^1^H time-domain nuclear magnetic resonance relaxometry (^1^H-TD-NMR). With this technique the water in the pores is the probe and thus does not need to be removed. As ^1^H-TD-NMR is sensitive to all protons present in the sample, it is also suited to measure nanoscale water-filled pores in the C-S-H structure [3,4] and to quantify the amount of hydrate phases developed and mixing water consumed during cement hydration [3,5,6].

Several NMR methods to study the pore space in hydrated cements have been proposed. McDonald et al. [4] used solid-echo (SE) pulse sequences to measure gradually dried cement pastes. They plotted the NMR signal intensity of these samples against the sample mass and found three distinct drying stages. Through a series of geometrical considerations and assumptions about the state of water, they acquired measures for two types of C-S-H pores. Muller et al. [3] used the fast exchange model of relaxation [7,8] to quantify the surface-to-volume ratio of the gel and interlayer pore space of C-S-H. This model requires an a priori unknown parameter, the surface relaxivity, which they calibrated from the *T*_2_ relaxation time measured at a sample equilibrated at low relative humidity conditions. They assume that in these conditions all surfaces in the sample were covered by a monolayer of water, which they assumed to consist of two monolayers of water molecules adsorbed to each layer of the interlayer space. In both methods described above, the calibration of the surface relaxivity was solely based on one measurement technique, ^1^H-TD-NMR, with assumptions about either the geometrical nature of the pore space or the physico-chemical nature of the pore surfaces. Cross referencing the findings with an additional measurement technique would help to check the validity of the methods.

In this work, we propose a new method to calibrate the system’s surface relaxivity based on a combination of SEM imaging and ^1^H-TD-NMR measurements. The SEM sample preparation adapted the argon broad ion beam (Ar-BIB) technique used for sandstones [9] for hydrated tricalcium silicate. High resolution SEM imaging and following image analysis was applied to prepared surfaces. The advantage of this method is that large surfaces (mm²) are sectioned without destruction of nanoscale structures. The achievable resolution for SEM imaging of porous materials is therefore independent of the sample preparation. It only depends on the imaging performance of the electron microscope used [9].

The method applied in the present study relies on image analysis of the interhydrate pore size. Thus, the surface relaxivity calibration was performed on the larger pores in hydrated tricalcium silicate (C_3_S) that should not have been altered due to drying of the sample. This new method was tested against, and compared with, the drying method presented by Muller et al. [3] assuming monolayer water molecule coverage in the interlayer space, the smallest pores in hydrated C_3_S. Both methods were conducted on hydrated monoclinic C_3_S pastes.

Electron-microscopic imaging and analysis techniques are advancing fast with increasing computing power and ^1^H-TD-NMR is established as a standard analysis method in the cement industry [10,11]. This ongoing development will increase the demand for measurement methods that put the acquired devices to use, such as the method presented in this work. The demonstrated pore space investigation will help in the design of new mortar and concrete formulations with enhanced physical properties due to an optimized pore space distribution.

## 2. Materials and Methods 

### 2.1. Materials

Commercially available tricalcium silicate (C_3_S) (Vustah, Czech Republic) of the MIII polymorph was used for the investigations. The chemical composition measured by X-ray fluorescence spectroscopy (XRF) is shown in Table 1. Phase purity of the C_3_S was checked by X-ray diffraction (97.6 wt.% crystalline C_3_S).

For all experiments, the C_3_S was mixed with deionized water and hydrated for 28 d in sealed containers. To obtain a completely water saturated microstructure, the samples for NMR measurements were additionally covered with a thin layer of saturated portlandite solution after one day of hydration.

### 2.2. Pore Size Evaluation

Pore sizes were determined by employing ^1^H-TD-NMR and a surface relaxivity calibration via two alternative methods, i) sequential drying of the C-S-H to a monolayer H_2_O coverage [3,12], and ii) combination of SEM image analysis and ^1^H-TD-NMR measurements. The ^1^H-TD-NMR measurements were performed on a Bruker minispec mq20 spectrometer operating at 19.95 MHz. The temperature controlled probe head, with a dead time around 5.4 µs, was set at 23 °C. *T*_2_ spin-spin relaxation measurements were performed using solid-echo [13] (also called quadrature-echo) and CPMG (Carr-Purcell-Meiboom-Gill) [14,15] pulse sequences. The *T*_2_ relaxation results were evaluated using Gaussian and exponential decay functions for the solid-echo measurements [13] and multiexponential fitting (sum of four exponential decay functions, Levenberg-Marquard fitting algorithm) for the CPMG measurements. The measurements were repeated twice using a total of three individually prepared samples.

The fast exchange model [7,8] gives a relationship between the surface-to-volume ratio (*S/V*) of pores and the resulting *T_1_* and *T*_2_ relaxation times,

(1) $$\frac{1}{T_{2}}\approx \frac{S}{V}\times \lambda ,$$

where λ is the surface relaxivity, which highly depends on the individual sample surface chemistry. This equation was used in both of the following calibration methods. For interlayer and gel pores, a planar pore geometry was assumed, where *S* = 2*A* and *V = Ad*. Thus *S/V* = 2/*d* for planar pores. For interhydrate pores a spherical shape was assumed, where *S/V* = 6/*d*.

#### 2.2.1. Determination of the Surface Relaxivity from Monolayer H_2_O Coverage

In order to reach a monolayer H_2_O coverage of the C-S-H interlayer pore space, 28-d hydrated samples were gradually dried in a laboratory furnace with temperatures increasing from 23 °C to 120 °C. Overall, 16 drying steps were made and the samples were measured in between drying steps using solid-echo and CPMG pulse sequences, as described above. The resulting *T*_2_ relaxation time for the C-S-H interlayer water at maximum drying conditions was combined with a water monolayer thickness of 0.28 nm [16], following Muller et al. [3], to calculate the surface relaxivity using Equation (1). This surface relaxivity was in turn used to calculate the *S/V* ratio and pore diameters of the water-saturated interlayer, gel, and capillary pore spaces, employing the individual *T*_2_ relaxation times measured for the non-dried samples.

#### 2.2.2. Combination of TD-NMR and SEM

To be able to acquire high resolution SEM images of the 28-d hydrated samples, argon broad ion beam sectioning was used for sample preparation. This preparation method avoids artifacts like scratches or smearing on the surface of the specimen, in comparison to the regular polishing. Furthermore, it is not necessary to embed the specimen in resin. 

The C_3_S hydration process was stopped after 28 d by immersing the specimen in ethanol (98%) for 20 min. Afterwards, the specimen was dried at 40 °C for several hours. A 7 × 3 × 5 mm^3^ piece was dissected. It was then milled in a Leica EM TIC 3X, an Ar-BIB milling system(Leica Microsystems, Wetzlar, Germany), using three ion beams and a stationary sample holder at 6 kV with a 2 mA gun current for 3 h followed by another 3 h treatment at 3 kV and 1 mA.

The specimen was then analyzed using a FEI Nova NanoSEM scanning electron microscope without a conductive coating. The images were acquired using the through the lens secondary electron (TLD SE) detector. Subjects of the analysis were mainly areas containing dense C-S-H phases with very little or no preparation damage. Since the specimen was highly sensitive to the electron beam and the thereby induced heat, low voltage, low current settings (2 kV, 22 pA) and a short observation time were mandatory to achieve high magnifications without affecting the pore structure. 

The image analysis was performed using the open source software Fiji [17] and self-developed macros, which can be found online [18]. A semi-automated process was used to segment the pores. Twenty-five images (a cumulative area of 192 µm²) with the same magnification (approx. 2.91 × 2.91 nm²/px) and containing mostly porous areas were analyzed. The images were optimized for segmentation (non-local means denoising, contrast enhancement) and the pores were segmented using the automatic thresholding method by Phansalkar et al. [19] utilizing modified parameters (radius = 6 px, *k* = 0.15, and *r* = 0.3, see elsewhere for details [18]). After the application of morphological operators (‘despeckle’ and ‘fill holes’) to denoise the binary images, the chord-length density function (CDF) [20](Chapter 2.5) of areas identified as pores was calculated in *x* and *y*-directions to improve the data density. While it is possible to calculate the pore size distribution (PSD) directly, the area analyzed was insufficient to determine a mean interhydrate pore size. Therefore, the CDF was determined as PSD. Nevertheless, it should be noticed that it is not trivially possible to convert the CDF to a PSD for non-circular polydisperse pores or particles. Therefore, the CDF might have led to a slightly translated (overestimated) interhydrate pore size. 

The mean interhydrate pore size was determined from the resulting pore size distribution. This was combined with the longest interhydrate *T*_2_ relaxation time, expected to correspond to the interhydrate pores, in order to calculate the surface relaxivity *λ* according to Equation (1). The surface relaxivity was then used again to calculate the *S/V* ratio and pore diameters of the interlayer and gel pore spaces for the samples.

## 3. Results and Discussion

**Figure 1:**
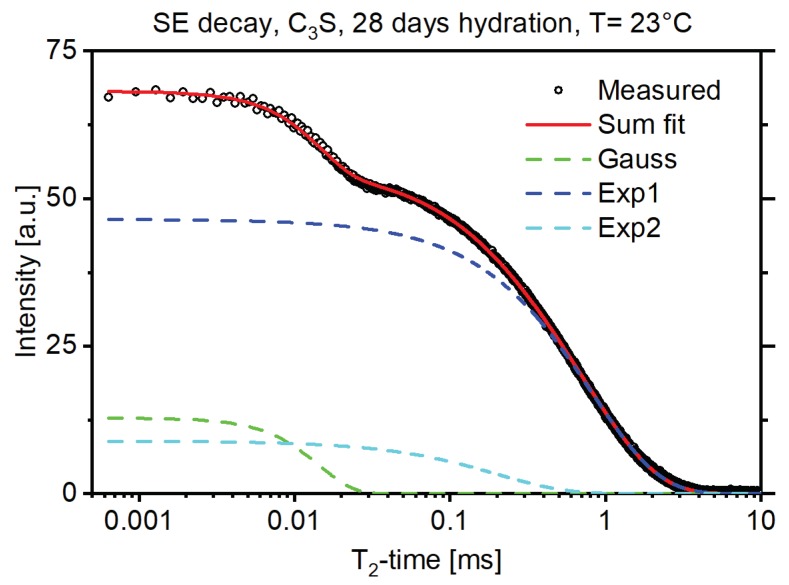
Solid-echo decay of a 28d hydrated C_3_S sample (black circles) with the Gaussian and exponential decay fits.

**Figure 2:**
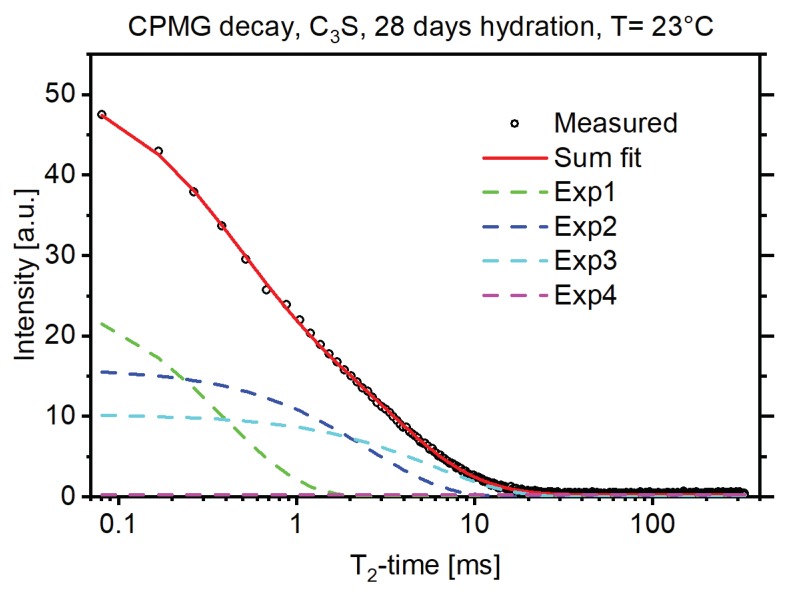
CPMG decay of a 28-d hydrated C_3_S sample (black circles) with the multiexponential fitting results using four exponential decay functions.

Figure 1 shows an exemplary solid-echo *T*_2_ magnetization decay of a 28-d hydrated C_3_S sample. The decay was fit with a Gaussian decay function to account for the protons rigidly bound, and two exponential functions for the more mobile protons in the sample [13]. In NMR *T*_2_ measurements rigidly bound protons, for example protons bound in a crystal structure, exhibited shorter *T*_2_ times while protons loosely bound, for example protons of the pore water, exhibited longer *T*_2_ times. The amplitude of the Gaussian fit thus corresponded to the protons bound in the crystal structure of the formed portlandite (CH) and the amplitude of the exponential fits corresponded to the protons in the C-S-H structure and the free water [3,5]. Figure 2 shows that this proton fraction can be further split into three major reservoirs, in accordance with the existing literature [3], by measuring the CPMG decay of the sample in combination with multiexponential fitting as described above. These reservoirs could be attributed to the protons bound in the C-S-H interlayer space (Exp1, green dashed line), the protons bound in the C-S-H gel pores (Exp2, blue dashed line), and the protons in interhydrate pores (Exp3 and Exp4, light blue and magenta dashed line). For interpretation of the references to color in the figures, the reader is referred to the web version of this article.

The final result of the solid-echo and CPMG measurements with the mean value for the three measured samples after 28 d of hydration are listed in Table 2. It was also noted that 18.4 at.% of the protons and thus 18.4 wt.% of the water in the sample was bound in the CH structure; 40.8 wt.% was bound in the C-S-H interlayer and 24.5 wt.% was bound in the C-S-H gel pores. The interhydrate pores contained 15.9 wt.% of the water in the sample. Additionally, Table 2 lists the mean value for the determined *T*_2_ times for each proton reservoir, which were used in the next sections to calibrate the surface relaxivity and to calculate the corresponding planar or spherical pore diameters.

### 3.1. Monolayer H_2_O Coverage Relaxation

The *T*_2_ relaxation time for the interlayer protons at high drying conditions was determined to be 310 µs. Considering planar pores, where *S/V* = 2/*d* according to Section 2.2 and a water monolayer thickness of 0.28 nm as half the distance for the planar interlayer pore space, Equation (1) delivered a value of 9.0 × 10^−4^ nm/µs for the surface relaxivity calibrated by this method. Calculated from this surface relaxivity, the pore diameters for the proton reservoirs, listed in Table 2 with their corresponding *T*_2_ times, were 0.72 nm for the C-S-H interlayer and 4.9 nm for the C-S-H gel pores considering a planar pore geometry. The calculated pore diameter for the interhydrate pores was 33 nm assuming a spherical pore geometry.

### 3.2. Combination of SEM and TD-NMR

**Figure 3:**
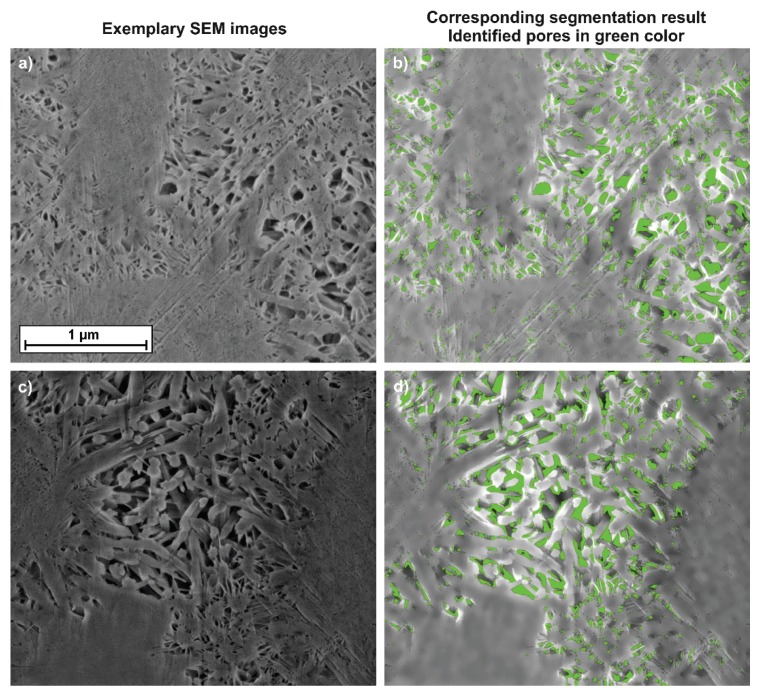
Two exemplary image segmentation results. The acquired SEM images (**a,c**) and the resulting segmentation with the pores in green (**b,d**). All images have the same scale bar.

Two exemplary high resolution SEM images of the 28-d hydrated C_3_S samples are shown in Figure 3a–d. The interhydrate pore space is clearly visible, as well as the overgrowing inner and outer product C-S-H phase. The two images on the left side show the acquired SEM images. Figure 3b and 3d show the image analysis results with the segmented pore space colored in green. It is obvious that segmentation of smaller pores (Figure 3b is more precise than for larger pores (Figure 3d).

**Figure 4:**
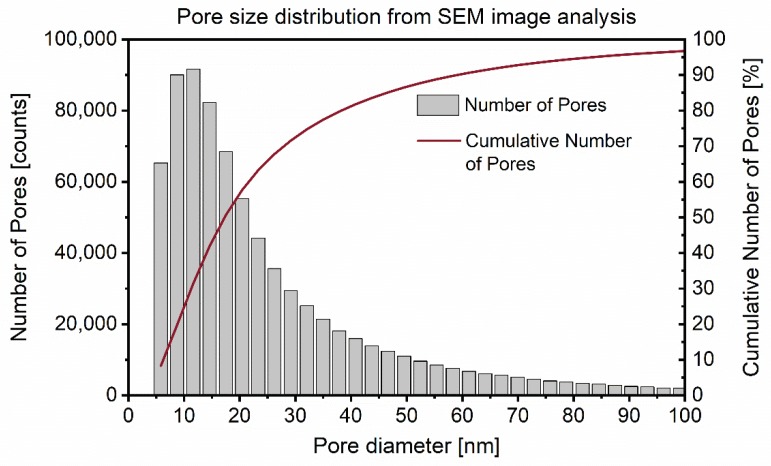
Interhydrate pore size distribution from automated image analysis of Ar-BIB milled 28 d hydrated C_3_S samples. The chord-length density function (CDF) was determined as pore diameter.

Figure 4 shows the resulting interhydrate pore size distribution derived from the segmented image analysis of the samples as described in Section 2.2.2. In this distribution, one bin spans 3 nm in size on the *x*-axis. All the pores with a diameter smaller than 6 nm are represented in the first bin. The second bin contains pores with between 6 and 9 nm diameters and so forth. 

The number of pores is plotted on the left *y*-axis in Figure 4. There was a high number of relatively small pores and the number of pores decreased exponentially with pore size. The mean pore size weighted by the total number of pores was 28.4 nm. The cumulative number of pores in % is shown on the right *y*-axis in Figure 4.

However, for the surface relaxivity calibration (Equation (1)) it is not the number of pores of different sizes, but rather the total pore surface and volume that have a direct influence on the proton relaxation. For pores with an increasing diameter, an increasing deviation from the assumed spherical pore shape and increasing interconnectedness was observed, leading to increasing underestimation of surface-to-volume ratio. Therefore, the calibration of the surface relaxivity was calculated based on the ratio of the sums of surface and volume of all pores smaller than 100 nm, attributing to 97% of the total pore count.

The resultant surface-to-volume ratio of 0.11 nm^-1^ was used for the surface relaxivity calibration. Considering spherical pores and the *T*_2_ relaxation time for interhydrate pores of 6089 µs, this resulted in a surface relaxivity of 1.49 × 10^−3^ nm/µs employing the relationship in Equation (1). According to the *T*_2_ times for C-S-H gel pores and the C-S-H interlayer shown in Table 2, this led to planar pore diameters of 8.1 and 1.2 nm as shown in Table 3.

### 3.3. Comparison of the Two Methods Employed for Surface Relaxivity Calibration

The main results of the two surface relaxivity calibration methods, the drying method assuming monolayer H_2_O coverage and the combination of SEM and NMR, are shown in Table 3. Considering the two completely different approaches, the resulting surface relaxivity values of 0.9 × 10^−3^ and 1.49 × 10^−3^ nm/µs were quite comparable. Consequently, the calculated pore sizes for C-S-H interlayer pores, C-S-H gel pores, and interhydrate pores were also comparable. Furthermore, the interlayer and gel pore sizes obtained in this study were comparable to the 0.94 nm and 3.1 nm reported by Muller et al. [3] for a hydrated white cement paste. Their *T*_2_ relaxation was faster due to a different chemistry of the samples with a surface relaxivity value of 3.73 × 10^−3^ nm/µs.

The surface relaxivity calibration through a combination of SEM image analysis and TD-NMR presented in this work confirmed the drying method proposed in the literature [3,7,8]. This was, however, only possible by employing a careful sample preparation routine presented in Section 2.2.2 as to not destroy the interhydrate pore space of the hydrated samples. It has to be noted that the presented new surface relaxivity calibration method relies on measured physical data, i.e., the pore sizes from SEM image analysis. No assumptions need to be made about the physico-chemical conditions of the C-S-H interlayer space, in particular the monolayer H_2_O coverage of the interlayer pore surface. Therefore, compared to the monolayer calibration method, the new method relies on fewer assumptions.

As the image analysis demonstrates (Figure 4), there was a wide range of interhydrate pore sizes spanning from around 6 nm to over 100 nm. Additionally, those pores were far from being perfectly spherical. Thus, the resulting values for pore diameters shown in Table 3 should be seen as approximate mean values and might not fully reflect the complex nature of nanoscale pore spaces. Recent advances in three-dimensional combined focused ion beam/scanning electron microscopy (FIB/SEM) sectioning and image analysis of hydrated cements are likely to enable a more detailed characterization of the pore surface, volume and shape in the future [21]. 

In line with previous authors, we chose the fast exchange model with a single relaxation time valid for all surfaces in the sample. Since it was sensitive to the surface chemistry, it may have been affected by the chemical composition and the phases present in the sample and may have been different in small and larger pores. Interestingly, since most phases other than C-S-H were crystalline without an internal porosity, they should have contributed to the surface of the larger pores and should therefore have had a stronger effect on the surface relaxivity of the new method. In the monolayer method, the contribution of the phases should be weighted by their contribution to the total surface. Thus, the surface relaxivity obtained by this method should be more characteristic for the internal C-S-H porosity. This could assign a physical meaning to the two different values of the surface relaxivity found. It would have been inappropriate to do so in our experiments due to the uncertainties of the averaging processes involved. More refined future experiments may indeed extend these ideas towards differentiating between the surface relaxivities in both pores.

## 4. Conclusions

- The new method for the NMR surface relaxivity calibration presented in this work relies on a combined analysis of SEM images and ^1^H-TD-NMR measurements.
- Argon broad ion beam sectioning allowed us to preserve and image the interhydrate pore space of hydrated tricalcium silicate with high resolution.
- The acquired results were comparable to the results obtained by an already established drying method, assuming monolayer H_2_O coverage.
- In contrast to this established method, the new method presented does not solely rely on NMR measurements and assumptions about the physico-chemical conditions of the sample surface.
- The physical parameters determined by this pore size characterization are important for models of the nanostructure of C-S-H, such as [22,23].
- Apart from being relevant for fundamental research, the method presented could also help in the design of new mortar and concrete formulations.

## Figures and Tables

**Table 1:** Chemical composition of the used C_3_S measured by X-ray fluorescence spectroscopy (XRF).

Oxides [wt.%]	C_3_S
SiO_2_	26.5
TiO_2_	0.03
Al_2_O_3_	0.34
Fe_2_O_3_	0.07
Mn_2_O_3_	0.02
MgO	2.02
CaO	70.3
Na_2_O	0.04
K_2_O	0.03
P_2_O_5_	0.13
LOI	0.46
∑ [wt.%]	99.8

**Table 2:** H reservoir distribution resulting from the evaluation of the solid-echo and CPMG decay results.

H Reservoir	H Fraction [at. %]	*T*_2_ [µs]
Portlandite (CH)	18.4 ± 0.3	14.1 ± 0.1
C-S-H interlayer	40.8 ± 0.2	407 ± 10
C-S-H gel pores	24.5 ± 0.2	2694 ± 110
Interhydrate pores	15.9 ± 0.3	6089 ± 151

**Table 3:** Surface relaxivity and pore sizes calibrated via monolayer H_2_O coverage and SEM–NMR combination compared to literature values.

Calibration Method	Surface Relaxivity [nm/µs]	C-S-H Interlayer [nm]	C-S-H Gel Pores [nm]	Interhydrate Pores [nm]
Monolayer H_2_O coverage	9.0 × 10^−4^	0.72	4.9	33
Combination SEM-NMR	1.49 × 10^−3^	1.2	8.1	54
From Muller et al. [3]	3.73 × 10^−3^	0.94	3.1	-
